# EID Photo Quiz

**DOI:** 10.3201/eid1412.081187

**Published:** 2008-12

**Keywords:** Photo quiz

**Figure:**
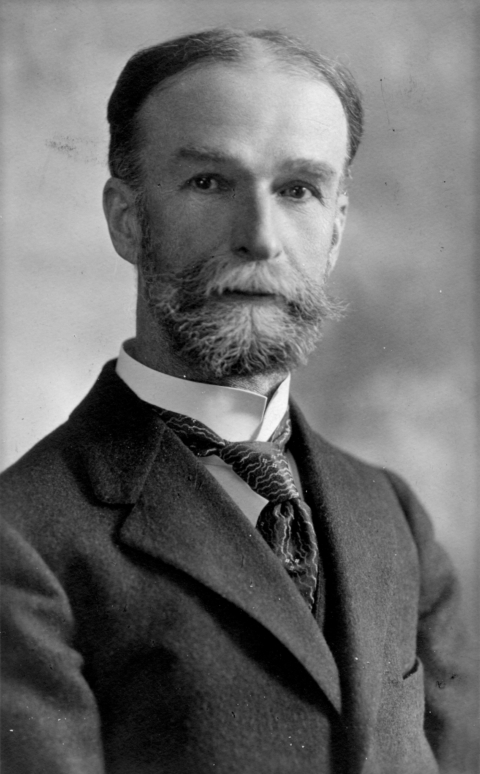
Who is he?

The theme of this month’s issue of EID is zoonotic diseases. Consistent with this theme, here is a photograph of a physician who greatly advanced medicine and public health through his investigations of diseases of animals. He and his colleagues were the first to show that insects can transmit disease.

## Who is he?

**A) Sir David Bruce**
**B) Robert Koch**
**C) Sir William Osler**
**D) Sir Ronald Ross**
**E) Theobald Smith**

Decide first. Then see companion article.

